# “I Somehow Survived… but I Will Never Do It Again”: Teachers’ Perspectives on Past and Future Educational Disruptions in Slovenia

**DOI:** 10.3390/ijerph22111740

**Published:** 2025-11-18

**Authors:** Urška Štremfel, Manja Veldin

**Affiliations:** Educational Research Institute, Gerbičeva ulica 62, 1000 Ljubljana, Slovenia; manja.veldin@pei.si

**Keywords:** teachers, work-related stress, occupational well-being, disruption, support

## Abstract

Five years after the COVID-19 pandemic, namely, the biggest disruption to education in the last century, this article provides insights into the consequences it holds for teachers’ well-being, their (non)preparedness for and support needed in any such future disruptions in Slovenia. By utilising the Job Demands–Resources Model, insights are provided concerning job demands (stress) and job resources (support) on different levels (individual, micro, meso, macro, chrono) of Bronfenbrenner’s ecological systems theory. The importance of complementing large-scale, representative, quantitative data (from the Responses to Educational Disruption Survey (REDS)) with qualitative data obtained from two focus groups comprising eight teachers in total is demonstrated to gain a comprehensive understanding of teachers’ well-being during educational disruptions. This study confirms that the intertwining of different levels in Bronfenbrenner’s socio-ecological system explains job demands (sources of stress) and job resources (support for teacher well-being) during the COVID-19 pandemic. The majority of stressors at the time of the pandemic were identified on the micro level, whereas sources of support were primarily located on the individual and meso (school) levels. For any future educational disruptions, however, the most significant sources of support for teachers’ well-being are expected on the macro level (system and society).

## 1. Introduction

Natural and social emergencies that disrupt education, such as health crises, earthquakes and floods, have significant impacts on the educational process. The COVID-19 pandemic (hereinafter, the pandemic) caused the most widespread and prolonged disruption in education over the last century. The unprecedented challenges it introduced to the educational environment have spurred extensive research and academic discussions (e.g., [1]) about its short- and long-term influences on the educational process and the implications for potential further disruptions to education (e.g., [2]). While much of this research has focused on students, studies on teachers’ work-related well-being during the pandemic remain relatively scarce [3]. However, existing findings (e.g., [4]) indicate that the pandemic profoundly altered teachers’ professional and personal lives, added to their work-related and general stress, and impacted both their personal and occupational well-being. It is a well-established finding that stress produces negative impacts on teachers’ well-being [5,6,7,8]. While the sources of teachers’ stress during the pandemic have been explored to some extent, research on the mechanisms that supported teachers in mitigating stress and enhancing well-being remains limited [9,10,11]. In a systematic review, the authors report a predominance of quantitative research on teacher well-being in this period (79.6%), with considerably fewer studies employing qualitative (9.7%) or mixed-methods (10.7%) approaches [12]. Further, few studies have concentrated on teachers’ perspectives, examining how challenges and support systems influenced their occupational well-being during and especially after the pandemic. Although research (e.g., [13]) suggests that challenges to teachers’ well-being continued throughout the pandemic, its long-term effects remain largely unknown [14,15,16,17].

Still, research suggests that critical life events can have a lasting impact on individuals and that adaptation may take many years, and in some cases might never happen [18]. Studying teacher stress and well-being during the COVID-19 pandemic from a retrospective perspective of 5 years, as provided in this article, is therefore important since it allows for analysis of how the pandemic affected teachers over time, beyond immediate reactions. It helps identify chronic stressors that have either persisted or intensified, as well as temporary or situational ones. As teachers’ coping strategies and well-being likely evolved in different phases of the pandemic, a retrospective lens helps to identify adaptive behaviours that can inform teachers’ professional development [19]. Retrospective studies accordingly add depth and context to existing findings, ensuring a more complete understanding of the pandemic’s impact and its implications for any subsequent educational disruptions. In 2025, marking the fifth anniversary of the COVID-19 pandemic, renewed policy and scholarly debates have emerged concerning the preparedness of countries and education systems for future large-scale disruptions. In this context, teacher stress and well-being have gained fresh attention, given their critical role in influencing teaching effectiveness and student outcomes (e.g., [11,20]). Chronic occupational stress, especially when compounded by insufficient institutional support and limited resources, can lead to professional burnout (e.g., [21]) and elevated teacher turnover rates [6,22]. Moreover, heightened teacher stress and burnout have been associated with increased classroom disruptions [23], weakened student–teacher relationships [24], greater student stress levels [25], and lower academic outcomes [23]. All these factors carry profound implications for public health. Teachers not only occupy a pivotal position within schools but also constitute a considerable segment of the national workforce. For example, in Slovenia in 2024, educators across the entire vertical of the education system accounted for 5.23% of the active working population [26,27]. The collective well-being of this occupational group, therefore, has population-level consequences that extend well beyond individual health outcomes [28]. Moreover, the (in)stability, (in)effectiveness, and (un)sustainability of the education workforce exert significant influence on student and community well-being, educational equity, and, ultimately, the long-term health of the population [29].

Education systems around the world responded to the COVID-19 disruption in diverse ways [30]. Slovenia offers a compelling case study because the pandemic triggered the country’s first large-scale shift to distance learning. Before this, Slovenian teachers had some (a little) experience with distance learning, though not with pandemic-forced closures. School closures were extended and implemented with little to no preparation, creating significant professional challenges for teachers [31]. At the time, there were virtually no national guidelines or training programmes available to support teachers while managing the disruptions to education. As a result, schools and individual teachers were left to develop their own responses to the evolving situation. Moreover, Slovenia still lacks a comprehensive strategy or policy framework for addressing future disruptions to education. While some countries, such as Ireland [32], developed national guidelines for maintaining teachers’ well-being during the pandemic, no such guidelines for teachers were published in Slovenia. This makes teachers’ experiences and perspectives particularly valuable for informing both school-level practices and national policy development. Notably, Slovenia is one of only two European countries that participated in the Responses to Educational Disruption Survey (REDS) [33]. However, broader and deeper research insights into teachers’ well-being during such disruptions in Slovenia remain limited, and existing studies often rely on non-representative samples.

### 1.1. Aim and Research Question

This paper aims to contribute to these scholarly discussions and address the identified research gaps by investigating: (1) how teachers in Slovenia perceived their work-related stress and the support available for their occupational well-being during the pandemic; (2) how the pandemic affected their (non)preparedness for any future educational disruptions; and (3) which types of support do they anticipate are needed in future educational disruptions. The goal is to answer the following research questions: (a) What were the primary sources of occupational stress for teachers during the COVID-19 pandemic on different levels of the socio-ecological system? (b) What were the main sources of occupational support for teachers during the COVID-19 pandemic on different levels of the socio-ecological system? (c) How can teachers be better supported during future disruptions to regular education on different levels of the socio-ecological system?

### 1.2. Structure

The paper is structured as follows. The introductory section outlines the research topic, identifies existing gaps, and outlines the study’s contributions, aims, and research questions. The subsequent part of the introduction provides the theoretical framework and an overview of relevant studies in the field. The second section describes the materials and methods employed, including the research procedure, participants, instruments, and analytical approach. The third section presents the quantitative and qualitative findings, organised according to Bronfenbrenner’s levels of the socio-ecological system. The fourth section discusses the principal findings and acknowledges the study’s limitations. Finally, the conclusion synthesises the main contributions of the research to scholarly debates and considers its implications for policy and practice.

### 1.3. Theoretical Insights

The study integrates two theoretical approaches. First, the Job Demands–Resources Model [34] is used to conceptualise the teachers’ work environment, identifying job demands (sources of stress) and available support mechanisms, whether physical, psychological, social, or organisational, that help mitigate their stress. In the second phase, these factors are categorised within Bronfenbrenner’s socio-ecological framework [35], which distinguishes five levels of influence on teachers’ functioning: the individual level (internal factors), micro level (students, classroom dynamics), meso level (colleagues and school leadership), macro level (educational policies, societal factors) and chrono level (changes over time).

The Job Demands–Resources (JD-R) model provides a dynamic framework for understanding how individuals navigate the balance between job demands and available resources. Employees (including teachers) can effectively manage their workload when job demands and resources are in balance. However, when job demands outweigh the available resources, stress arises due to the perceived imbalance [36,37]. Subjective appraisals play a crucial role in determining which resources are most effective in managing specific demands, given that individuals assess and utilise resources based on their perceived relevance and adequacy [38]. In this regard, each teacher’s unique appraisals of their work demands and resources determine the degree to which they are at risk for stress [39]. The model recognises that this balance is not static but shifts over time, influenced by changing work conditions and personal capacities [36].

A key strength of the JD-R model is its ability to respond to varying job demands across different contexts. It allows for the inclusion of unique factors relevant to specific work environments, offering flexibility and adaptability. This makes the JD-R model a valuable tool for understanding how employees cope with work demands and sustain their well-being over time [34]. In this respect, the value of the JD-R model has already been shown while studying the dynamic relationship between teacher stress and well-being (e.g., [40,41,42]) and support mechanisms during the COVID-19 pandemic (e.g., [43,44,45,46,47,48]). As the balance between job demands and resources [36] was disturbed during the pandemic [49], job resources buffering the job demands caused by the pandemic were requested [50,51]. In Italy, for example, job resources fostered teachers’ personal resources (self-efficacy and resilience), and teachers’ personal responses contributed to lower emotional exhaustion [45]. Similarly, various job resources such as support from colleagues and previous information and communication technology use contributed to lower stress and less exhaustion, while improving job satisfaction in teachers in Germany [48]. In addition, some authors explain that teachers experienced the COVID-19 pandemic very differently and identified three subgroups of teachers in this sense [7]. One subgroup of teachers (“Worried and stressed”) was characterised by high levels of stress, high job demands, but low job resources, and little fulfilment of their basic psychological needs. A second group of teachers (“Relaxed”) consisted of teachers who experienced relatively low levels of stress, were satisfied with their jobs, and had medium levels of job resources combined with somewhat lower job demands. And third, the largest group of teachers (“Happy work-a-holics”) reported both high job demands and high job resources, and had experienced relatively little stress and high job satisfaction. Their findings reveal the interplay of individual and other levels of the socio-ecological system in explaining teachers’ well-being during the pandemic.

The JD-R model’s sensitivity to shifts in the demands–resource balance over time [36] aligns with Bronfenbrenner’s ecological systems theory [35], which stresses the interplay between individuals and their environments. From Bronfenbrenner’s perspective, subjective appraisals occur within the teacher’s microsystem, where direct interactions with colleagues, students, and administrators shape their perceptions of job resources. On the mesosystem level, the JD-R model’s adaptability reflects how teaching demands vary across interconnected settings, such as schools and policy environments. The macrosystem further influences this dynamic by embedding broader institutional and policy-level factors that affect job demands and resource availability. On the macrosystem level, cultural expectations shape what is perceived as a resource or a demand, reinforcing the JD-R model’s contextual flexibility. Bronfenbrenner’s ecological systems theory has therefore been applied to the study of teachers’ well-being, stress, and mental health both before the pandemic (e.g., [52,53,54]) and during it (e.g., [13,55]). Table 1 presents a synthesis of findings from existing studies that investigated the sources of teachers’ stress and the support they received during the pandemic. These sources are classified according to the various levels in Bronfenbrenner’s socio-ecological model. This structured overview provides a systematic insight into prior research in the field, facilitating a comparison of our findings with those of earlier studies.

Similarly, fewer themes were identified while describing three key areas of teachers’ needs during the pandemic [71]. In the area of teachers’ work–life balance and working from home, the need for a clearer line between the two and the need for fixed working hours and a fixed daily structure were most frequently expressed. In the area of teaching and interaction with students and parents, more contact with and visibility of students was the most exposed. In the area of school management and colleagues, the need for more or better school management, including more guidelines, clarity, and guidance from the school management, was most frequently indicated.

In relation to current/future support needed, authors [16] report that teachers have called for changes in the post-pandemic scenario, particularly focusing on continuous professional development (specific training in digital pedagogy and technological tools), enhancing institutional conditions (reducing bureaucratic overload, introducing clear rules on the functioning of online platforms, the creation of new assessment criteria adapted to remote teaching), and securing teachers’ and students’ access to adequate resources (appropriate technological devices and stable Internet connections).

While research on job demands (a source of stress) and job resources (a source of support) during the pandemic has been relatively extensive, much less attention has been paid to teachers’ preparedness and the support they might need in the event of future educational disruptions on different socio-ecological levels. These gaps reveal there is a need to pay closer attention to what is still required and how we can prepare teachers to face future educational challenges. This paper seeks to address these outstanding questions identified in the existing literature.

## 2. Materials and Methods

### 2.1. Procedure

This paper forms part of the basic research project “Effects of the COVID-19 Pandemic on Schooling, Teachers and Students: Well-Being, Teaching and Learning”, funded by the Slovenian Research and Innovation Agency (2022–2025). An explanatory sequential design was employed to triangulate quantitative and qualitative data, in which the qualitative phase—teacher focus groups—was used to explain, elaborate, and contextualise the findings of the Responses to Educational Disruption Survey (REDS) [72].

The quantitative analysis draws on descriptive statistics from the REDS [33]. The mentioned survey was a large-scale comparative study conducted by UNESCO (United Nations Educational, Scientific and Cultural Organization) and the IEA (International Association for the Evaluation of Educational Achievement). The study sought to examine how education systems across different countries responded to the challenges of ensuring educational provision at the time of the COVID-19 pandemic. In 2020–2021, REDS collected data from 11 education systems around the world (Burkina Faso, Denmark, Ethiopia, India, Kenya, Russian Federation, Rwanda, Slovenia, United Arab Emirates, Uruguay, Uzbekistan), encompassing a representative sample of eighth-grade students, teachers, and principals. The qualitative component consists of two focus groups with teachers from Slovenia, conducted in February 2025. Data collection in the focus groups was carried out after obtaining informed consent from all participants. Both focus group sessions were conducted online using Zoom. Each session was facilitated by a single interviewer, accompanied by a technician, and followed a pre-prepared protocol. Each focus group meeting lasted approximately 90 min.

While both (quantitative and qualitative) approaches have been widely used to study teachers’ responses to the pandemic, to our knowledge, their combined application remains unexplored. The strength of this mixed-methods approach lies in its complementarity. The REDS data, based on a representative sample, allow for the findings to be generalised for the entire population of Slovenian teachers. Meanwhile, the focus groups provide deeper insights into teachers’ responses in the REDS, offering a more nuanced understanding of the COVID-19 pandemic’s impact on their occupational well-being.

### 2.2. Participants

The paper analyses responses from 1422 eighth-grade teachers in Slovenia. The sample is representative of the population of teachers who teach grade 8 students. The estimated population is 5868 teachers in the targeted grade [33]. The qualitative component comprised two focus groups conducted in February 2025, with a total of eight participants. The first focus group was made up of three participants, while the second group had five participants. Participants in the focus groups were randomly selected from the list of REDS school coordinators in Slovenia and invited to participate by email. See Table 2 for demographic data of both samples.

In the first step, from the list of 136 REDS school coordinators, those who were not engaged in teaching activities during the pandemic (e.g., school leaders, school counsellors) were identified. In the second step, these non-teaching coordinators were excluded from the sample, resulting in a refined list of 29 REDS coordinators who were actively involved in teaching during the pandemic. In the third step, 14 teachers—7 for each of the two planned focus groups—were randomly selected from this pool using the Research Randomizer software (https://www.randomizer.org/, accessed on 15 January 2025). In the fourth step, the selected teachers were invited to participate in the focus groups. Although consent was obtained from all 14 invitees, the final number of participants was lower (*n* = 8), as several were unable to attend due to emergent work obligations (e.g., substitution duties). This outcome reflects the broader contextual challenges associated with teacher shortages in Slovenia. The final sample consisted of eight female teachers representing various career stages and teaching different subjects (Slovene, Mathematics, English, Art, and Science) across eight primary schools in Slovenia. Exact demographic data were not collected due to the small sample size in order to protect participants’ anonymity and ensure confidentiality.

### 2.3. Instruments

The 2021 REDS collected comprehensive data on participants’ backgrounds and their perceptions of various aspects of life affected by the COVID-19 pandemic. The variables encompass school organisation, instructional practices, challenges and changes in teaching and learning, as well as the well-being of both students and teachers, including measures implemented to support well-being in schools. In this study, only data from the teacher questionnaire are included. We used two questions examining teacher well-being and perceived support during the disruption caused by the pandemic (Q16 and Q18; see [73]). Teachers rated their level of agreement with statements about their well-being and the support they had received using a four-point Likert scale ranging from 1 (strongly agree) to 4 (strongly disagree).

The focus groups protocol included six questions for teachers regarding sources of stress during the COVID-19 pandemic, sources of support in that time, the (lack of) preparedness for future educational disruptions, and the types of support needed in any future disruptions. When responding to all questions, participants were encouraged to reflect on factors at all levels of Bronfenbrenner’s ecological systems theory [35].

### 2.4. Analysis

The analysis of the REDS data was based on secondary data obtained from the national REDS report [74], rather than the original REDS study dataset. While the REDS dataset is publicly accessible (https://www.iea.nl/data-tools/repository/reds, accessed on 16 February 2025), re-analysis of the original data was unnecessary as the required information was already provided in the national report. Specifically, our analysis focused on the percentages of teachers who agreed or strongly agreed with statements relating to teacher well-being (as outlined above). For the purpose of this study, these items were subsequently categorised as sources of stress or support across the levels of Bronfenbrenner’s ecological systems theory [35], using an independent coding process conducted by two researchers. The focus groups were audio-recorded and transcribed using Sonix software. Directed content analysis [75] was used to examine teachers’ sources of stress and support during the COVID-19 pandemic, the (non)preparedness for and support needed in potential future educational disruptions. As for the REDS data, the analysis was guided by the Job Demands–Resources (JD-R) model [36] and Bronfenbrenner’s ecological systems theory [35]. The coding process followed a structured approach: (i) deductive coding—initial coding categories (source of stress, source of support, (non)preparedness, support needed) were defined in advance based on the JD-R model and organised according to the different levels in Bronfenbrenner’s socio-ecological system (individual, micro, meso, macro, chrono); (ii) inductive refinement–new themes that emerged from the data were incorporated into the coding scheme to capture context-specific insights; (iii) validation—two researchers independently analysed and coded the text, then met to discuss discrepancies, refine category definitions, and align their coding decisions, which added to the consistency and validity of the analysis by integrating multiple perspectives and reducing individual bias; and (iv) thematic analysis—the coded data were then analysed to identify key patterns related to sources of teachers’ stress and support, as well as (non)preparedness and support needed in any future educational disruptions.

## 3. Results

### 3.1. Teachers’ Stress Sources and Well-Being Sources as Reported in the REDS

**Figure 1:**
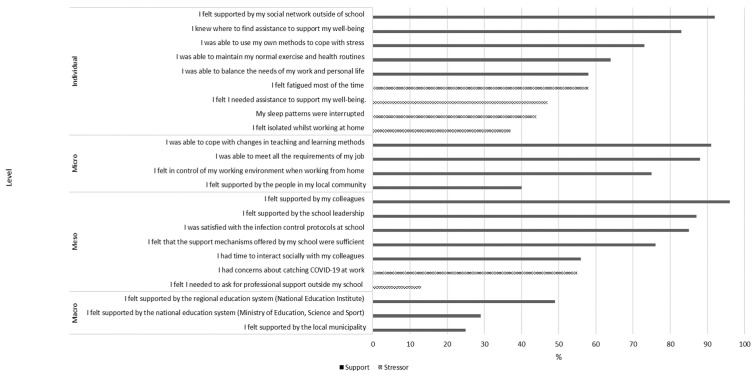
Perceptions of stressors and sources of support during the COVID-19 pandemic in Slovenia across socio-ecological levels (REDS data; [74]).

The REDS results revealed the teachers’ stress and well-being in Slovenia to be a pressing issue [33,74]. Figure 1 is based on REDS data and shows the share of teachers who reported (the percentage of teachers who agreed or strongly agreed) having experienced various sources of stressors and support during the pandemic, organised by different levels in Bronfenbrenner’s ecological systems theory.

At the individual level, the majority of teachers reported being able to rely on their own coping strategies (73%) and maintain healthy routines (64%). They knew where to seek support for their well-being (83%), and nearly half of them reported needing it at the time (47%). Although nearly all felt supported by their social networks (92%), almost half of them felt isolated while working from home (37%). Around half the teachers reported having experienced sleep disturbances (44%) and frequent fatigue (58%), and had trouble balancing their work and personal life (58%). At the micro level, most teachers indicated that they were able to meet job demands (88%) and felt in control of their work while teaching remotely (75%). Further, almost everyone reported being able to cope with the ongoing changes to their teaching practices and interactions with students (91%). However, less than half of them felt supported by people in their local community (40%). Social support was stronger on the meso level, with over 90% of teachers highlighting strong support from colleagues as a key protective factor. Nonetheless, only half of them (56%) reported having time to engage with their colleagues socially. The majority also felt supported by the school leadership (87%) and expressed satisfaction with the infection control protocols (85%) and school-based support mechanisms (76%). Still, some teachers had to seek professional help outside of school during this time (13%), and around half voiced concerns about contracting COVID-19 at work (55%), revealing persistent sources of stress. At the macro level, fewer teachers perceived support from national (29%) and regional (49%) education systems. Moreover, support from the local municipality was also perceived as scarce (25%). This suggests a notable gap in systemic-level support during the pandemic.

### 3.2. Teachers’ Stress Sources and Well-Being Support as Reported in the Focus Groups

Below, the results of the focus groups (teachers’ sources of stress, resources for well-being, future support needed) are organised according to Bronfenbrenner’s levels of the socio-ecological system. See Table 3 for an overview.

#### 3.2.1. Individual Level

The participating teachers reported large differences in stress levels, depending on their personal circumstances, such as being parents of small children or living alone. Teachers (Teachers 1, 2, 3, 4, 5, 7, 8) who had small children were more stressed about workload, timelines, maintaining a work–life balance, and sharing space, a computer, and an Internet connection with other family members. On the other hand, one teacher (Teacher 6) who was living alone reported experiencing less stress in managing the increased workload and work demands. They recognised that their personality influenced their stress control. “At the beginning, enthusiasm and positivity solved many problems” (Teacher 1). They also acknowledged the importance of internal resources for balancing stress. For example, Teacher 3 explained: “The first problem was with me, how to clarify, where do I set the boundaries, where do I set the amount of learning material? It was always to come from myself. If I were able to balance myself, then I would be able to successfully transfer this pattern to other areas”. The importance of self-help was also recognised by Teacher 2: “You had to help yourself to survive”. They also used different opportunities to relax in nature (Teacher 4, 5, 7), spending time with the family and cooking (Teacher 7), and self-development opportunities available in that period, such as various self-help groups (Teacher 1).

All participating teachers felt the most prepared for a similar situation in terms of developed digital competencies and digital materials required for teaching at a distance. “I feel well-prepared for using digital technology for teaching./…/Digital skills and prepared teaching materials for online teaching would make teaching in repeated situations less demanding” (Teacher 8). The participating teachers (Teachers 1, 3, 4, 5, 6) reported being less prepared in the psychological field (their own and their students). “If it happened again, I would certainly go into it with some concern in my mind that I would not become burned out again” (Teacher 5). “I had such a very strong, difficult personal experience in that period that I think if it happen again, I would resign irrevocably. I am certainly the least prepared in the psychological field. Also, in terms of supporting students with psychological difficulties in that period” (Teacher 1). “Professionally, it would work, technically it would work, but at least I feel qualified in contact with parents and in psychological support for children” (Teacher 3). “A student had a panic attack during a class lesson online. And then I saw how we were unequipped for these things, and you cannot even react, or maybe you react in a way that would be the least desirable and necessary, and then a lot can come out of that” (Teacher 1). “We are not prepared to monitor a pupil’s well-being remotely, to communicate with parents in this area and to get the right information about the pupil’s well-being. Many things can happen during a prolonged absence” (Teacher 1). “You cannot be prepared for the difficult psychological problems children have when they return to school after a long absence” (Teacher 1). Some participating teachers reported being more prepared for some aspects of preserving their well-being. “We would probably introduce a little distance between us so that we are not always available as we were then. We should probably make some distance here, so that we have those limits a little bit” (Teacher 7). “I now have a clearer idea of who is responsible for the student being in class. I would be much less bothered from that point of view. And I would be able to deflect the shifting of responsibility” (Teacher 1).

#### 3.2.2. Micro Level

Among micro-level factors, the participating teachers (Teachers 1, 2, 3, 5, 6, 8) most frequently mentioned their care for (vulnerable) students as a source of stress during the pandemic. “For me, the most stressful thing was wanting to be able to give as much as I could to the children, so that they could get something from the classroom” (Teacher 8). Regarding inequalities in Internet access and equipment among students, Teacher 5 explained that some families with more children only had one computer, and some children were therefore learning on phones, which is not good for their development. Teacher 5 explained, “A lot of people were learning in small flats. No Internet connection. No computers. The school and the Ministry provided them, but it took (too) much time”. Teacher 1 stated: “I believe that equal conditions and the inclusion of all students in the same way are my priorities as a teacher in the classroom”. The participating teachers (Teachers 1, 2, 3, 5, 8) also felt stressed because of the preoccupation with the well-being of students. Teacher 8 noted, “It was hard for me to accept the rules about distancing, and it was hard for me to watch my children having a hard time, too”. More specifically, Teacher 2 explained, “The data that 15% of pupils do not have a single cooked meal at home when schools were closed was distressing and burdensome for me, as I had not thought about that before”. Another important issue and source of stress identified was the mental health of students. Teacher 3 reported that about 10% of students were ‘lost’ due to mental health issues or not being motivated for work. Concerning care for equal learning opportunities and the well-being of students, the participating teachers (Teachers 1, 3, 4, 6) found the role of the class teacher to be the most demanding. “I have to say that there was a lot of pressure on the class teachers to be the front line. In fact, we were the ones who had to somehow reach out to the pupils and the parents and retain them” (Teacher 1).

The participating teachers (Teachers 1, 2, 3, 5, 6) also reported the non-responsiveness of some students (and parents) to learning at a distance as an important source of stress. For example, Teacher 5 estimated that fewer students were actively doing their schoolwork than happens with regular schooling. Teacher 2 explained that the students’ response was 40%, and Teacher 3 estimated that 50% of the students were actively participating. Some teachers (Teachers 1, 5, 6) reported that, in this regard, the main source of stress for them was not having a clearly defined and communicated role concerning who was responsible for their attendance. Teacher 1 explained, “At our school, there were two groups of teachers: those who said, ‘Everything is prepared; whoever wants to participate can do so. It is accessible to everyone, and now it’s up to them and their parents if they don’t take advantage of it./…/But I was on the other side. I somehow felt responsible even for those students who would miss out on everything”. Teacher 5 noted that she was working hard to make sure students did everything and were active. Similarly, Teacher 6 explained, “As a class teacher, I was more stressed because there was more stress since individual pupils had problems with motivation, and I was also more in contact with parents about individual situations”.

The findings indicate mixed experiences regarding cooperation with parents. Some teachers (Teachers 3, 8) described communication with parents as a source of support. “They were supportive; discussions with them revealed that they were facing the same problems as we were in that period” (Teacher 3). “Some parents contacted us to thank us for our efforts with their children” (Teacher 8). However, teachers (Teachers 1, 4, 5, 6, 8) also found communication with parents to be demanding and stressful. “We had very poor communication with the parents. Parents here are generally very unresponsive to anything to do with education in our area, and that period was no exception” (Teacher 1). “Our efforts were not as appreciated by some parents for the work that went into it”. “Some parents complained about why pupils had to work so much” (Teacher 8). They also reported challenges with entering the student’s home environment and the parents being present. “When you teach remotely, parents are also present. It’s different when you have an adult audience. You have to get used to that” (Teacher 5). “Actually, we were entering our students’ home environment through distance learning. I remember one student, at the time of her oral exam, she was in the room, and her father was sleeping on the bed, having returned from the night shift. I didn’t feel good, how not good the children felt?” (Teacher 4).

With respect to preparedness for any future disruptions to education, on the students’ level, they (Teachers 1, 3, 4, 5) felt the students’ digital competencies are a potential (future) issue. “We try to keep students acquainted with digital technology in case of a similar situation. With a measure of caution for what lies ahead” (Teacher 1). “Despite what these students have learned about technology, we still find time and once again that they do not really know how to use it for the right purposes. Even though we teach them this every year, it is a problem, and it could be a problem” (Teacher 4). “Students’ digital literacy is not as high as it could be. There is still work to be done” (Teacher 3). They (Teachers 4, 5) also identified difficulties with connecting with deprivileged children and their parents. “Establishing a connection with migrant students and parents who do not speak Slovenian. This is not established yet. It was also very difficult the first time for teachers”.

#### 3.2.3. Meso Level

All participating teachers reported various forms of collegial support for controlling their stress and supporting their well-being in the pandemic. Professional support among teachers of specific subjects was particularly evident. “I would also like to praise the maths team because we helped each other a lot. Otherwise, we were very much on our own” (Teacher 7). “Within the group of Slovenian teachers, we agreed who would do what kind of video or game to make it a bit easier” (Teacher 5). “In foreign languages, my colleagues and I helped each other. We didn’t have any beginners; we all had at least 10 years of work experience, and we took care of ourselves, our health, and well-being. That’s why we always held our meetings outdoors. We would meet outside to get some fresh air and take care of ourselves, so we weren’t just sitting in front of computers typing on keyboards” (Teacher 3). In contrast, teachers without specific subject teacher support reported a lack of it. “I’m an artist, so I’m the only one at the school, so I was really more on my own for everything” (Teacher 4). The participating teachers (e.g., Teacher 2) also mentioned how materials prepared with colleagues at the time are still very useful and valuable today. “When it was the coronavirus, all the teachers kind of got together and were ready to help each other, and were on our phones a lot. And it helped a lot with the stress, because you had people who were in a very similar situation as you” (Teacher 6). The collegial support on the school level was also exposed. “On the school level, for those of us who knew more about digital tools, we had a course for colleagues. So, there were five of us per Zoom session and we practised together, worked together and so on” (Teacher 5). “The yoga teacher offering a yoga course twice a week to relax, to stretch and provide instructions for exercises during teaching. This contributes a lot to well-being” (Teacher 2). “Those colleagues who had so much goodwill, optimism and energy kept the rest of us, who were perhaps a bit more at the bottom, relatively up” (Teacher 1).

The size of one’s school was identified as a source of stress as well as a protective factor. While a teacher from a large school (Teacher 2) reported the huge amount of work (e.g., teaching 300 students in that period also required checking their school projects and homework, responding to questions and e-mails, monitoring the level of their activity), they (Teachers 2, 3, 5, 7, 8) also identified the size of their school as a protective factor in terms of teachers’ collegial help in discussing new standards, preparing materials, as well as sharing tasks and subjects. A teacher from a small school (Teacher 1) was alone in performing these tasks and therefore felt more responsible, unsure and stressed. Although adopting teaching materials was not mentioned as a high stressor, the teachers pointed out that the amount of materials that exist varies significantly between different subjects. While in some subjects (e.g., English) a lot of online materials were already available (Teachers 1, 3, 6), in others (e.g., Slovenian, Science, Maths, Art) teachers (Teachers 2, 4, 5, 7) had to prepare them by themselves. Yet, as indicated above, the cooperation among subject teachers was exposed as important for controlling workload in larger schools.

The school management was also identified as giving important support for teachers’ well-being by almost all participating teachers (Teachers 1, 2, 3, 4, 5, 6, 8). “On the school level, we were well-organised, thanks to the school leadership” (Teacher 3). Teacher 8 reported feeling well-supported through regular discussions and consistent encouragement from the young headmistress, on whom the staff could always rely. “I would say 100%, just thank you. I can only praise the management, the head teacher and the vice-head teacher. I had a good experience./…/As I live in a very small flat in which it is difficult to draw a line between private and professional life, the school leadership allowed me to perform working tasks in the classroom, without any contact, of course” (Teacher 6). On the school level, frequent regular meetings for planning, evaluating work, and solving problems were identified as an important aspect of support for teachers’ well-being. Unity among school personnel regarding goals, expectations, and standards—along with frequent meetings and ongoing problem-solving—was found to support teachers’ well-being, although it could also be a source of stress when it involved lowering standards. The participating teachers (e.g., Teacher 8) also appreciated the accessibility of technology and the possibility of borrowing it from school for smooth work at home. Also much appreciated by the participating teachers was the support of a computer technician, “Especially in the 2nd wave, when part of the classroom was at school and part at home, it was very valuable” (Teacher 2). It was also missed when it was absent. “Our computer guy was on sick leave a lot, so we were on our own for everything” (Teacher 5).

In the absence of strict national regulations, the schools were quite autonomous in how they organised the teaching and learning process. “There was no incentive, not even from the ministry, on how to do something. We had a free hand, but I don’t know if that was a good thing at the time” (Teacher 8). While Teacher 3 reported that clear school rules on the number of hours to be taught online made the teaching process smoother, Teacher 8 reported that the freedom of teachers caused inequalities between teachers at the same school and is also a source of stress. “In our case, no teacher was forced to teach remotely. Older teachers just sent things via email. I did all my lessons via Zoom and was very tired at the end of each lesson. What I missed was that everyone was the same, that they had the lessons in the same way” (Teacher 8).

Depending on the school and its size, school psychologists were available to teachers (e.g., a large school; Teacher 3). Even though they reported that school psychologists also had a heavy workload, and in some schools mostly focused on the students’ and not the teachers’ well-being. “The counselling service worked mainly with pupils. They were the ones who got the attention, more than the teachers” (Teacher 7). “If you go there on your own to ask for help with a problem, then they are there for you. Otherwise, they don’t have the time or the will to deal with each teacher individually” (Teacher 7). “They have the will, but not the time. They were overloaded at work with several other obligations” (Teacher 4). “I do not perceive this as their task; they were more focused on students and family problems in that period” (Teacher 6).

All participating teachers appreciated the availability of school counsellors’ support during the COVID-19 pandemic and in regular schooling, but due to their several other obligations estimated it to be insufficient to meet all teachers’ well-being-related and other needs (during the pandemic and in any future disruptions). They referred to a kind of supervision. “I noticed that I am not as skilled in the area of communication. I don’t always find the answers to certain questions, and I ask myself if I have reacted correctly. In that sense, I lack the support of having someone to say this is what I did right and this is what I did wrong, to seemingly guide me” (Teacher 1). “In our case, the counselling service participates in a supervision group where they compare their experiences and the problems they are facing. It would help me as a class teacher to have a support system outside the school on how to deal with things” (Teacher 5). Despite the participating teachers reporting their increased levels of digital competencies they gained in the pandemic, they estimated that technical (computer) support would still be crucially needed in any future disruptions to education. This was particularly exposed by older teachers (Teacher 8).

#### 3.2.4. Macro Level

Despite the participating teachers (e.g., Teachers 1, 3) generally expressing an understanding that policy responses came with a time delay, they revealed that, in some cases, they led to the non-motivation of students to attend the online classes. “First, there were no active online courses; we lost some students, and motivation failed. Support from the system (e.g., computer support, teaching advice) always came unintentionally with a delay, because that is the nature of the work” (Teacher 3). Since the system was unprepared for the disruption, in many cases, the lack of online teaching at the very beginning caused the loss of a connection with the students, who had already lost their motivation to learn when the online system was established (Teachers 1, 3).

The participating teachers (Teachers 4, 5, 7) were very reserved concerning the support received from the Ministry of Education. “Leadership was right up my alley. But I didn’t feel any other major help. Not even from the Ministry. No, they did nothing of the kind. I think they just lowered the number of evaluations. Otherwise, I don’t know that there was anything else concrete. I didn’t feel that this was the case” (Teacher 4). “If I think back, I don’t know, but was there any support from the Ministry? I did not feel at the time that we were supported anywhere. We were basically teachers ourselves, on all sides. As far as the school management and the collective were concerned, you could help” (Teacher 7). “We were pretty much left to ourselves and our own organisation. So, it was really very tiring” (Teacher 5). In addition, the national protocols for testing for coronavirus were very open and needed to be adapted on the school level. Here, there were multiple different interpretations, also between parents, which caused a lot of stress in that period (Teachers 5, 6). The participating teachers (Teachers 2, 4, 7) also felt limited in terms of support from the National Education Institute. “In study groups at the National Institute for Education, you were acknowledged, which application to use” (Teacher 1). “We expected more, perhaps from the National Education Institute. Maybe they could have sent us some more materials and so on” (Teacher 7). “I think after it was over, we were getting different materials” (Teacher 7). “After the pandemic, I was attending the course at the National Education Institute, and I felt well in stress control. But this was conducted when the pandemic was already over” (Teacher 4).

The (negative) perception of the teaching profession in wider society was identified as a source of teacher stress. “It has often been said that we teachers are only at home, that we do nothing, that the students learn on their own” (Teacher 7). “In public and in general, the emphasis was on the pupil, not so much on the teacher. As adult employees, we were partly overlooked here. Because we are expected to do our job, and the focus was on how we were going to help the pupils get through this, how we were going to help the parents get through this, rather than what we were going to do for ourselves” (Teacher 6). However, the participating teachers appreciated the support available in society for self-development during that time period. “In society, there were a lot of sources for self-help freely available, very well presented, clear, and transparent” (Teacher 1).

Regarding policy support, the participating teachers in particular reported on those aspects needed in possible future disruptions. While teaching at a distance, they would appreciate “more detailed instructions from the Ministry on how to do things, not just prohibitions on what not to do” (Teacher 8). They mentioned the testing for coronavirus in schools by teachers and other educational professionals as one of the most pressing issues. “Teachers were the healthcare professionals when testing students for COVID-19, and if a test was positive, a psychologist. This could be better regulated” (Teacher 4). “We were not trained and responsible to do this. We would need support here if it were to happen again” (Teacher 3). Student assessment was also exposed as a pressing issue. “I see the problem mostly in students’ assessments. If we remember back, that was quite a huge problem. Let’s say that this situation happens again and lasts a long time. We cannot imagine such a remote assessment” (Teacher 6). “As far as the student assessment is concerned, it seems to me that it was and is a very unrealistic picture. There have been no developments on the system level since then”. They also suggested that the back-to-school rules (a reduced number of students in class, eating lunch in the classroom, the class staying in the same classroom the whole day, etc.) could be better, more tailored to the actual situations, and should allow for more flexible implementation, given that not all schools have the same conditions. This would also make teachers feel less burdened by the requirements (Teachers 4, 5). As they were overwhelmed with taking attendance and delivering lessons, and the students’ level of motivation for learning was low during the pandemic, some participating teachers (e.g., Teacher 1) also suggested an external (national) motivator to motivate students to learn remotely, either in general or at the start of the lessons. Instead of solely formal circulars, they also mentioned the value of a more personal and motivational approach by policymakers, which would support teachers in difficult times (e.g., live online teacher webcasts by policymakers). The value of a more personal approach is also evident in the supervision groups proposal, in which participating teachers would like their experiences and troubles on the grassroots level to be heard by policymakers at the Ministry or the National Education Institute (Teacher 5).

In terms of continuous professional development, they referred to the need for courses in the relational communication fields, like dealing with difficult students, such as: “In particular, the difficulties of the pupils, the difficulties of the parents, or working with the parents, suddenly become more difficult because the situation is more intense. This is the area that would cause me the greatest stress if it happened again” (Teacher 5). However, the feeling of helplessness is evident with regard to the possibilities of overcoming such situations. “The most stressful thing for me would be classroom work. And the parents. It was quite bad with them, let’s say, the threats to the press. I don’t know if you can ever be prepared for that” (Teacher 4). Notwithstanding that they recognised the increased levels of continuous professional development programmes in the field of stress regulation on the national level, not one of the participating teachers attended it on their own initiative (Teacher 5).

The findings on systematic preparedness are best summed up in the view of the following teacher. “I agree that a lot of materials are not available. There was also a lot of teacher training afterwards. As for being systemically ready for it, we are not./…/There will be expectations of parents and children that everything is well prepared and will run smoothly. But it is not prepared”. Teacher 4 added that, since it will be a repeated situation, expectations will also be higher and harder to achieve.

#### 3.2.5. Chrono Level

The COVID-19 pandemic itself represented a huge change, which, according to the participating teachers, created the following sources of stress. The high level of uncertainty was frequently identified as a significant source of stress, primarily due to the novel nature of the situation and the unpredictability of policy responses, which were subject to frequent, often daily, changes.

All participating teachers also reported the heavy workload in that period as an important source of stress. “If you are 100 percent present in class, you were 200 percent present at that time” (Teacher 5). “During the pandemic, my phone rang constantly. In fact, I was never really free” (Teacher 5). “I think I was at my peak throughout this period” (Teacher 3). “I think I was at my maximum during the whole period”/…/”Some older teachers gave up because of the situation, workload and adaptations needed” (Teacher 3). “It drove me to burnout. And I know many who, like me, burned out at that time because it was too much” (Teacher 5). “It was an interesting experience, but I would never repeat it” (Teacher 3). “I hope I never have to do it again because I really don’t want to” (Teacher 5). “I just think it is essential that we all want it not to happen again” (Teacher 5).

In terms of the chrono level, we can also refer to changes in the perception of COVID-19’s consequences from a distant point in time and the long-term COVID-19 consequences that the participating teachers observed. They reported the long-term consequences of COVID-19 on students’ knowledge and some students’ mental health. “We are still figuring out which generation is the corona generation, and there are still deficits in the basic concepts. Also, because some of them got tired of the work back then, and it is still there” (Teacher 5). “It seems to me that we have heard, seen and experienced so much about this (the psychological problems of pupils) that now, at the slightest sign, at the slightest discomfort that arises in lessons, teachers react much more immediately than they would have done before this period, because the experience has simply been very severe” (Teacher 1). They also experienced hard situations with particular students. “She became depressed, was hospitalised, had difficulties, and in the long run was recovering basically until the ninth grade” (Teacher 3).

## 4. Discussion

The pandemic had a significant impact on teachers’ work and personal lives, increasing their stress levels and posing challenges to their overall well-being [5,6,7,8]. The same was true in Slovenia, where the REDS findings identified teacher stress and well-being as a key concern [33,74]. To complement these results, we conducted focus groups 5 years after the pandemic began to gain a more nuanced understanding of teachers’ experiences. These retrospective insights provide a deeper understanding of the pandemic’s impact [19] and inform policy responses to potential future disruptions to education [16,76]. Drawing on Bronfenbrenner’s socio-ecological model [35], the analysis explores job demands (sources of stress) and job resources (supports for teacher well-being) during the pandemic, as well as teachers’ preparedness and needs in the face of possible future disruptions.

When job demands exceed the resources available, teachers experience increased stress, which (if prolonged and unsupported) can lead to burnout and higher turnover [6,21,22,34,45,77]. As reported by other authors [49] and as shown in Table 1, the balance between job demands and resources was disrupted globally during the pandemic. Teachers reported a decrease in resources as they ‘climbed’ the levels of the socio-ecological system. A similar trend is seen in our results, in both the REDS data and the focus groups (see Figure 1 and Table 3). The focus groups exposed the problem of burnout among the teachers and their colleagues (two teachers), which they attributed to the intense workload and changing working conditions during the pandemic. This aligns with the REDS data that show that 37% of teachers in Slovenia felt isolated whilst working at home, and around half the teachers reported constant fatigue (58%) and sleep disturbances (44%), with some even seeking professional help (47%). These results are consistent with the findings of authors who found that, although the average level of emotional exhaustion was moderate, over 20% of teachers reported having experienced a high level of emotional exhaustion [71]. Further, the majority of teachers reported an increase in emotional exhaustion and experienced the pandemic as harmful. Similar results from other authors show that the majority of teachers felt very or extremely stressed and that more than half felt very or extremely burned out [78]. These results are concerning since high levels of stress and emotional exhaustion in teachers have been shown to diminish their sense of agency [79], reduce their ability to provide creative learning opportunities [80], and have a negative impact on student outcomes [13,23,25].

### 4.1. Sources of Stress on Different Levels

When examining sources of stress, stressors are recognised on all levels of the socio-ecological system (Table 3). However, more than half can be attributed to the micro and macro levels. On the individual level, teachers who participated in the REDS reported significant personal stressors (e.g., fatigue), which are similar to the personal issues reported by other researchers (e.g., [16,58]). The teachers included in the focus groups did not identify physical health and safety as a primary source of stress, despite this being demonstrated by other international research (e.g., [13]). They did, however, express concern about their home environment (e.g., living arrangements and home resources) (seven teachers). Moreover, they reported difficulties with maintaining work–life balance and dealing with the increased workload (seven teachers), as also found in other studies (e.g., [13,14,16,55]).

The participating teachers reported many stressors on the micro level related to students and parents. Care for students was exposed as one of the most pressing issues by almost all participating teachers (six teachers) (see Table 3). This conforms with other research findings that one of the most prevalent concerns among teachers was student engagement, and teachers had to devise new strategies to motivate students, hold them accountable for their learning, and provide support in remote or hybrid settings [81,82]. Nevertheless, most teachers in the REDS reported having coped well with ongoing changes in their teaching practices and interactions with students (91%), meeting all the job requirements (88%), and feeling in control of their working environment (75%) (see Figure 1).

The care for students’ well-being as a pressing issue and an important source of stress also corresponds with other REDS findings (p. 145, [33]), in which 80% of the teachers who taught their class remotely during the COVID-19 disruption reported they provided support or information about emotional well-being to some or to a large extent, 45% of them also provided information about access to welfare services. Teachers in the focus groups also reported having to navigate students’ broader social contexts and home environments (five teachers). Challenges in communication and engagement (five teachers), along with unclear responsibilities (three teachers) and the transfer of (some of) the educational role to parents (five teachers), emerged as substantial sources of stress in this period, corresponding to findings in other studies [14,43]. The microsystem revealed a strong commitment to students’ well-being (six teachers), which, while a source of professional identity, also became a significant emotional burden (see also [83]), particularly in the absence of equal access to learning resources and student motivation.

On the meso level, around half the teachers (55%) included in the REDS voiced concerns about contracting COVID-19 at work, highlighting this as a persistent source of stress, as also reported by others [14]. Although teacher autonomy was recognised during the pandemic as contributing to positive outcomes such as improved performance [84], higher job satisfaction [85], and reduced burnout [85,86], it is worth noting that some of the participating teachers (three teachers) primarily experienced it as a source of inequality in working conditions and, thus, stress.

On the macro level, the teachers’ responses were characterised by disappointment with the lack of timely and concrete systemic support (six teachers). The public’s underappreciation of teachers’ efforts (three teachers) and vague national protocols (four teachers) further added to the stress and uncertainty. The policy and systemic challenges shown in our study are consistent with findings of other studies, which identified inconsistency in public health orders and their implementation [14,87], unrealistic expectations regarding the safety measures required to prevent the spread of the virus [13,14,88], and the lack of clarity and support from the authorities [13]. Overall, society portrayed the profession negatively in the media, as reported in other studies [55,58,83].

On the chrono level, all participating teachers reported sources of stress linked to the changes brought about by the COVID-19 pandemic. The observed decline in student knowledge (three teachers) and mental health (four teachers) (see also [89]) is a ‘new’ stressor for teachers in Slovenia. The high level of uncertainty, long-lasting overwork, and fatigue have gone untreated and led to burnout, as mentioned above. Similar concerns about accumulated demands have been reported by other authors (e.g., [58,71,90,91]). It is also clear that the different waves of the pandemic introduced new stressors over time. In the first phase, the novelty of the situation and resulting uncertainty emerged as key stressors (see also [92]). During the second wave, the primary source of stress was linked to national health regulations and their practical implementation (see also [65,83,93,94]).

### 4.2. Sources of Support on Different Levels

Research shows that perceived support for mental health and well-being is one of the strongest predictors of workplace well-being among teachers (e.g., [14]). During the COVID-19 pandemic, teachers reported having experienced strong social support across the individual, micro, and meso levels, particularly from their immediate social circle, colleagues, and school leadership. This aligns with Bronfenbrenner’s assertion that the most immediate social contexts, such as the self and close interpersonal relationships, have the greatest influence on individual functioning, including teacher well-being [95].

On the individual level, the focus group findings pointed to the importance of both external (e.g., time with family) (seven teachers) and internal coping strategies (e.g., emotional self-regulation) (four teachers) in managing stress. Similarly, the majority of REDS participants reported being able to rely on their own coping mechanisms (73%), maintain healthy routines (64%), and access support when needed (83%), most often through their personal social networks (92%). Other studies also identified psychological skills such as emotional regulation and self-efficacy as important protective factors (e.g., [45,58,60,61]), although social support on this level was less frequently emphasised. While skills and prior experience with distance learning were shown to buffer stress in other studies (e.g., [51,62,63,64]), this aspect was not prominently reflected in our data.

On the micro level, teachers in the focus groups (three teachers) identified supportive parents as the only meaningful source of support. This observation contrasts with other studies, where students were seen as the primary source of support, providing a sense of meaning and reward (e.g., [11]). However, in a study on remote learning during the lockdown, authors also noted an “increased level of trust between parents and teachers” (p. 17, [68]), highlighting the importance of strengthened parent-teacher communication in this period.

On the meso level, collegial collaboration and supportive school leadership emerged as key protective factors. Teachers especially valued the cooperation within subject-aligned teams (six teachers) and the support of school leaders (seven teachers). These findings are consistent with prior research showing peer and leadership support as crucial for teacher well-being during the pandemic (e.g., [14,48,66,67]). REDS data from Slovenia support this, with 96% of teachers having felt supported by colleagues, 87% by school leadership, 49% by the regional education system, and only 29% by the national education system. Similar patterns were observed internationally [14], where teachers reported the strongest support from peers, followed by school administrators, and the least from national authorities. Our findings confirm that at the time of the pandemic, teachers needed and received social, technical, and pedagogical support [96]. Although the research shows the importance of school psychologists’ support in maintaining teachers’ well-being [39], the participating teachers reported that these were received to a very limited extent due to the school psychologists’ preoccupation with other tasks in that period, including students’ issues and well-being. Still, co-workers and school leaders (meso level) played an important role in teachers’ well-being during these extraordinary times (e.g., [11,48,62,97]).

On the macro level, both the focus groups (six teachers) and the REDS data (see above) indicated that system-level support was perceived as limited, pointing to the need for more robust national and regional structures to assist teachers during disruptions to education. In the absence of adequate institutional support, teachers often turned to publicly available self-help resources (two teachers) and relied heavily on their social networks (seven teachers).

Although previous studies rarely identified support on the chrono level, all eight participating teachers emphasised that the digital skills acquired and lessons learned during the pandemic have become valuable long-term resources. These experiences were seen as supportive in navigating current challenges and adding to preparedness for any future disruptions.

### 4.3. Limitations and Future Research Directions

Although combining large-scale survey data with in-depth qualitative insights strengthens the study’s contribution, there are limitations that should be noted. For instance, the REDS relied on self-reported data from eighth-grade teachers in Slovenia, which means that it might not capture the experiences of individuals working on different educational levels, countries, or in other cultural contexts. While the focus group participants provided valuable insights, they were drawn from a small, non-representative pool of eight REDS school coordinators, and thus cannot be considered representative of the entire population. They may also have introduced a selection bias towards more engaged teachers. Further, the focus groups were conducted in 2025, several years after the peak of the disruption caused by the pandemic, which may have influenced the participants’ recollections and interpretations of events. Finally, since the quantitative and qualitative data were collected at different times, integrating the findings provides complementary perspectives rather than direct, time-aligned comparisons. Although the article draws primarily on existing research from the Anglo-Saxon world and empirical research conducted in Slovenia (a Central European country) and may therefore overlook broader perspectives on educational disruption in the Global South, its findings, given the largely universal nature of educational disruptions, may nevertheless hold relevance in a wider global context. To capture a broader range of perspectives, future research should include teachers from various educational levels, countries, and cultural contexts to enhance generalizability. Including a wider range of participants could also provide a more nuanced understanding of teacher well-being and support during and after disruptions. Finally, collecting quantitative and qualitative data concurrently or longitudinally would allow for more robust findings.

### 4.4. (Non)Preparedness and Required Support for Future Educational Disruptions Across Levels

While the sources of stress and support during the pandemic are well-documented, empirical evidence on teachers’ preparedness and systemic support needed on different levels of the socio-ecological system in potential future disruptions remains scarce. Teachers in the focus groups highlighted the importance of additional support, particularly on the meso and macro levels, to better manage similar situations in the future. Addressing these needs is vital for building more resilient and equitable education systems and ensuring sustainable change in education [90].

On the individual level, teachers stressed the importance of establishing clearer boundaries between their professional and personal lives since blurred roles during the school closures contributed to greater stress and burnout (four teachers). On the micro level, teachers identified the need for clearer guidelines about their roles and responsibilities during an educational disruption (six teachers) (for similar findings, see [43,93]). Improved role clarity for all participants in the learning process (teachers, students, parents) would help to reduce confusion and emotional overload in times of crisis.

On the meso level, teachers emphasised the importance of peer collaboration (seven teachers) and effective school leadership (seven teachers). They called for formal systems of peer support and supervision (four teachers), access to psychosocial services (two teachers), and targeted training with respect to different aspects of teachers’ well-being (e.g., stress management, communication) (six teachers). They also noted the need for technical support (two teachers) and pedagogical tools (three teachers) for effective remote teaching, alongside valid assessment methods (four teachers) and support for disadvantaged or migrant students (six teachers). This is in line with findings from others [16], where the participants advocated changes in the post-pandemic scenario, especially focusing on continuous professional development, enhancing institutional conditions, and securing access to adequate resources.

On the macro level, where teachers reported inconsistent and limited systemic support during the pandemic, they stressed the need for clear, realistic, and flexible national guidelines (four teachers), as well as more humanised and motivational communication from policymakers (one teacher). Importantly, teachers (four teachers) expressed a desire for more direct dialogue with education authorities to make sure that policies are grounded in the realities of everyday teaching.

Finally, on the chrono level, all eight teachers underscored the pandemic’s lasting impacts and the critical need for system-wide preparedness for future disruptions to education. They acknowledged the value of the digital competencies (eight teachers) and coping strategies (four teachers) developed during the crisis and called for sustained investment in student mental health (six teachers), digital literacy (five teachers), and inclusive support for marginalised learners (five teachers). Notably, all eight participating teachers assessed their digital skills as adequate for delivering remote instruction, suggesting a significant increase in their digital readiness throughout the pandemic (see also [16]). However, while teachers reported improved digital preparedness, they also observed a critical gap in their ability to support psychological well-being, both their own and that of their students (six teachers). This underscores the need for future support systems that extend beyond technical capacity to include structured training in mental health, access to supervision, and well-designed crisis communication protocols tailored to the educational context. In sum, these findings call for a more integrated and proactive policy approach that strengthens not only the digital but also the emotional and relational competencies of educational workers. In building systemic resilience, it is imperative to recognise and support the human dimensions of education.

## 5. Conclusions

As the probability of a severe pandemic in the coming decades is relatively high [98], and potential natural disasters (e.g., floods, wildfires, earthquakes) and military conflicts could bring similar impacts and disrupt education, education systems, including teachers, school leaders, and policymakers, need to be well prepared for such situations (e.g., [99]). The lessons learnt from the COVID-19 pandemic, as the largest disruption to education seen in the last century, are vitally important in this regard. Psychological and occupational well-being is a crucial factor in teachers’ ability to function effectively in both regular and disrupted educational environments. As research indicates, teachers’ well-being directly influences their work performance (e.g., [100]) and, in turn, impacts students’ learning outcomes (e.g., [101]). Without adequate support, work-related stress can lead to teacher shortages, declining mental health, and, ultimately, poorer educational outcomes for students [13]. All these have important implications for public health [28]. Given these implications, teachers’ well-being during and after educational disruptions warrants greater research attention—an area that remains insufficiently explored. In the paper, we focused on teachers’ views on sources of stress and support during the pandemic, along with their (non)preparedness and the support needed in any future disruptions to education.

Our study confirms the intertwining of the different levels in Bronfenbrenner’s socio-ecological system in explaining the job demands (sources of stress) and job resources (support for teacher well-being) during the COVID-19 pandemic. The results reveal the multifaceted nature of teacher stress and resilience at the time of the COVID-19 pandemic, as well as future needs in such situations. Teachers’ experiences span across all ecological levels, with each level contributing both stressors and supports. As Teacher 3 explained, “I think it was a kind of intertwining of different environments, different experiences, and you were learning to swim along the way”. However, they reported different levels of support on a particular level. “The pupils were stressed, we (the teachers) were stressed. Thank God for the support of the leadership, but we were not so much supported by higher instances” (Teacher 8). These findings indicate that support on the meso level, such as from colleagues and school management, can ease teachers’ stress, which chiefly originates from the micro (students) and meso (school) levels. They also suggest that meso-level support can somewhat compensate for the lack of support on the macro (system) level. They confirm how subjective appraisals play a crucial role in determining which resources are most effective in managing certain demands since individuals assess and utilise resources based on their perceived relevance and adequacy [34,38]. Our findings therefore demonstrate the importance of combining the Job Demands–Resources (JD-R) model [36] with Bronfenbrenner’s socio-ecological systems theory to provide a more comprehensive and coherent conceptual framework for understanding teachers’ well-being—by capturing both their demands (sources of stress) and resources (sources of support) during past and future disruptions to education.

The paper shows the importance of a mixed-method approach for examining teachers’ well-being during educational disruptions. It emphasises the value of complementing the quantitative, representative findings of the REDS on teachers’ well-being in Slovenia with qualitative data from focus groups. The focus groups identified additional sources of stress and support on all levels of Bronfenbrenner’s socio-ecological system in areas not addressed by the REDS (see Figure 1) and provided deeper insights into teachers’ preparedness and needs in any future educational disruptions. This further reveals the importance of conducting in-depth national studies on teachers’ well-being during educational disruptions, particularly given the diverse ways in which education systems around the world responded to the COVID-19 crisis (e.g., [30]). For instance, while teacher autonomy during the pandemic was viewed as supportive of well-being in several education systems (e.g., [70,84,85,86]), the findings presented in this paper reveal that Slovenian teachers largely perceived such autonomy as a source of stress. Furthermore, our results indicate that fear for physical health and safety did not emerge as a significant stressor among teachers in Slovenia, unlike findings reported in other national contexts. These safety protocols were thus perceived primarily as a source of stress, without recognising that they might also function as a support mechanism, reinforcing teachers’ feelings of safety in the workplace. This not only highlights the distinctive characteristics of the education system but also introduces a new dimension to the development of Job Demands–Resources (JD-R) theory, particularly regarding the conditions under which a certain factor is perceived as a resource (source of support) or a demand (source of stress). Moreover, it recognises the importance of incorporating teachers’ reflections on their profound experiences (particularly the sources of stress and support) during the COVID-19 pandemic into a comprehensive and in-depth evaluation of the education system’s past response (e.g., [102]). Understanding pandemic-related challenges and support systems from teachers’ perspectives provides a critical foundation for developing evidence-based recommendations to better support them in their current roles and in any future disruptions to education. This understanding highlights teachers’ perceived (un)preparedness and identified needs, which are essential for informing policy measures aimed at enhancing their well-being [12], building resilient education systems (e.g., [103]), and supporting sustainable change in education [90].

The findings strongly suggest the need to address teacher well-being in Slovenian schools. This could serve for their functioning in regular teaching as well as preparedness for any further disruptions in education when their well-being is particularly affected. As job resources (support received on different levels in Bronfenbrenner’s ecological system) act as a buffer against the negative effects of job demands (sources of teachers’ occupational stress) [21], it is important to systematically provide them, both on the meso and macro levels. The results imply that promoting within-school collaboration and providing teachers with the foundation to establish and build collegial relationships [100], identified as important for mitigating teachers’ stress and maintaining their well-being during the pandemic, should be further systemically supported. In addition, the current study reveals that, in particular, teacher professional development in the wider field of teachers’ well-being should be strengthened. Even though in Slovenia some programmes for enhancing teachers’ social and emotional competencies have already been verified (e.g., HAND in HAND; [104]) and evidence-based recommendations for their exploitation have been provided (e.g., [105]), it is important to ensure their broader exploitation on the system level. It must be noted that the responsibility for promoting teacher well-being does not rest only on teachers themselves (individual level) or on the schools and school leaders (meso level) as identified in our study, but also on policy and society as a whole (macro level). Since teachers play a pivotal role in the sustainable development of societies [12], policy should provide support for teacher well-being by employing key preventive public health strategies, such as investing in teacher support systems, providing evidence-based professional development, and carrying out proactive crisis planning. This study highlights the importance of taking such a systemic approach to teacher well-being by integrating interventions at micro (students), meso (school), and macro (system) levels. Addressing well-being at all levels enables education systems to enhance teachers’ professional functioning, protect student outcomes, and foster resilience in the face of future disruptions. It is necessary to ensure that, in the event of future educational disruptions, teachers do not simply report, “I somehow survived… but I will never do it again”, and instead feel empowered to address the challenges already identified and those that may emerge. Policies that support teachers’ well-being are seen as a crucial step forward and an important preventive public-health strategy.

## Figures and Tables

**Table 1:** Teachers’ job demands and resources during the COVID-19 pandemic.

Level in Bronfenbrenner’s Ecological System	Job Demands/Stressors	Job Resources/Supports
**Individual**	**Psychological and physical health challenges:** Anxiety [13,56,57]; health struggles and personal issues [16,58]; work and personal life balance [14,16,55,59]; feeling unsafe in the workplace [14] **Professional demands:** Adapting teaching to distance learning [55]; lack of ICT skills; increased and additional workload [13,55]	**Psychological characteristics:** Resilience, positive self-perceptions (e.g., competence), emotional regulation [45,60]; high locus of control and self-efficacy [61]; coping strategies [58] **Skills and experience:** Pre-existing ICT skills [51,62]; self-efficacy in distance teaching [51]; previous distance learning experience [63,64]
**Micro (students, classroom)**	**Concerns about students and families:** Concern for others [58], especially students’ academic achievement, well-being, home life, and behaviour [13,16,65]; increased needs for various types of support to families [14]; missing connection with students, unable to meet their needs and reduced learning time [14]	**Meaning:** Students as a source of meaning and reward [11]
**Meso (school leadership)**	**Organisational demands and conditions:** Lack of institutional support; professional demands, work responsibilities, and instructional expectations from leadership [13,43]; poor working conditions [16]; dissatisfaction with school safety measures [14] **Health risks at school:** Exposure to unvaccinated children [14]; being at risk of infection at school	**Support from school leadership and peers:** Support received by the colleagues and school management (either material, organisational, or emotional [14,48,66,67]; acknowledging and supporting well-being and encouraging work–life balance (School wellness initiatives and school safety protocols (feeling safe)) [14], School computer technology [62]
**Macro (system/society)**	**Policy and systemic challenges:** Inconsistency in public health orders and implementation, feeling unsupported by the government [14]; unrealistic COVID safety expectations [13,14]; delayed vaccine access—no protections as afforded to other essential workers [14], standardised testing pressure; rapid changes in teaching conditions [14] **Communication and public perception:** Lack of clarity from government and school leadership leading to variability in educational action plans [13] and exacerbating the challenge of the shift and feelings of uncertainty [68,69]; negative media portrayal [55,58]	Work autonomy and social support [58,70]
**Chrono (temporal changes)**	**Accumulated demands:** Learning new technologies [14]; uncertainty, increased workload, and managing multiple roles simultaneously [58]	

**Table 2:** Study sample characteristics.

Characteristics	Quantitative Sample	Qualitative Sample
Sample size (*N*)	1422	8
Gender (% female)	78.30%	100%
Subjects taught	Various subjects	Slovene, Mathematics, English, Art, Science
Years of teaching experience	19.9% ≤ 10 years 34.4% = 11–20 years 45.8% ≥ 21 years	Representing various career stages

**Table 3:** Sources of stress, support, and future needs identified by teachers categorised according to Bronfenbrenner’s socio-ecological system.

Level in Bronfenbrenner’s Ecological System	Sources of Stress	Sources of Support for Well-Being	Future Support Needs
**Individual**	- Personal circumstances (e.g., parenting, living alone)	- Internal coping (e.g., self-regulation, optimism)	- Boundary-setting and professional role clarity
	- Workload and work–life balance	- Time spent in nature and with the family	
	- Limited home resources (sharing space, computer, and Internet with the family)	- Use of self-help strategies	
**Micro (students, classroom)**	Emotional burden of student care (concern for (vulnerable) students, their well-being, and mental health) - Unequal access (devices, Internet, food, study space) - Low student/parent engagement - Ambiguity in responsibilities (attendance, motivation) - Burden of being a class teacher - Challenging communication with parents	- Supportive parents	- Clearer role definitions (guidelines on roles/responsibilities)
**Meso (school leadership)**	- Isolation among teachers in less-represented subjects - Lowering educational standards - Unequal workload distribution (due to the lack of national regulation and school-level coordination) data	- Collegial support - Subject-specific collaboration - Supportive school leadership (e.g., regular meetings, accessibility of technology, school space) - School-based initiatives (e.g., yoga, outdoor meetings) - School psychologists (mostly focused on students’ and not teachers’ well-being) - Subject-related resources developed during the pandemic	- Peer support and supervision systems - Accessible psychosocial services for staff (mental health support for teachers, as well as students) - Training in stress management and self-care - Training in communication and relational skills - Technical (information technology) support - External (national) motivators for student engagement in distance learning - Tools and guidelines for online well-being monitoring - Valid remote assessment practices - Digital skills training for students - Support for migrant students and families
**Macro (system, society)**	- Lack of timely guidance from authorities - Size of school (bigger—more work; smaller—less collegial help) - Uneven availability of teaching materials between different subjects - Limited support from authorities (e.g., the Ministry of Education, the National Education Institute) - Public devaluation of the teaching profession - Poor coordination of protocols for COVID-19 testing	- Size of school (bigger, more collegial help) - Publicly available self-help resources	- Clear, feasible and flexible national guidelines (e.g., testing for coronavirus, tailored back-to-school rules) - Humanised and motivational support from policymakers - Direct dialogue opportunities with policymakers
**Chrono (temporal changes)**	- Long-term overwork and fatigue - Unpredictable and changing rules - High level of uncertainty - Declining student knowledge and mental health	- Acquired digital skills - Lessons learned from past experiences	- System-level preparedness for future disruptions - Flexible assessment procedures - Support for student mental health and digital literacy - Special focus on marginalised students (e.g., migrants, low socioeconomic status)

## Data Availability

The REDS datasets supporting the conclusions of this article are openly accessible and can be downloaded as public-use files from the IEA website: https://www.iea.nl/data-tools/repository/reds (accessed on 16 February 2025). The data from the focus groups is stored and available upon reasonable request from the authors of the article.

## Abbreviations

The following abbreviations are used in this manuscript:

COVID-19	Coronavirus disease 2019
IEA	International Association for the Evaluation of Educational Achievement
JD-R	Job Demands–Resources Model
REDS	Responses to Educational Disruption Survey
UNESCO	United Nations Educational, Scientific and Cultural Organization
